# A Review of *Swertia chirayita* (Gentianaceae) as a Traditional Medicinal Plant

**DOI:** 10.3389/fphar.2015.00308

**Published:** 2016-01-12

**Authors:** Vijay Kumar, Johannes Van Staden

**Affiliations:** Research Centre for Plant Growth and Development, School of Life Sciences, University of KwaZulu-NatalPietermaritzburg, South Africa

**Keywords:** biological activity, conservation, medicinal plant, *Swertia chirayita*, traditional medicine

## Abstract

*Swertia chirayita* (Gentianaceae), a popular medicinal herb indigenous to the temperate Himalayas is used in traditional medicine to treat numerous ailments such as liver disorders, malaria, and diabetes and are reported to have a wide spectrum of pharmacological properties. Its medicinal usage is well-documented in Indian pharmaceutical codex, the British, and the American pharmacopeias and in different traditional medicine such as the Ayurveda, Unani, Siddha, and other conventional medical systems. This ethnomedicinal herb is known mostly for its bitter taste caused by the presence of different bioactive compounds that are directly associated with human health welfare. The increasing high usage of *Swertia chirayita*, mostly the underground tissues, as well as the illegal overharvesting combined with habitat destruction resulted in a drastic reduction of its populations and has brought this plant to the verge of extinction. The increasing national and international demand for *Swertia chirayita* has led to unscrupulous collection from the wild and adulteration of supplies. The aim of this review is to provide a synthesis of the current state of scientific knowledge on the medicinal uses, phytochemistry, pharmacological activities, safety evaluation as well as the potential role of plant biotechnology in the conservation of *Swertia chirayita* and to highlight its future prospects. Pharmacological data reported in literature suggest that *Swertia chirayita* shows a beneficial effect in the treatment of several ailments. However, there is lack of adequate information on the safety evaluation of the plant. The pharmacological usefulness of *Swertia chirayita* requires the need for conservation-friendly approaches in its utilization. Providing high-quality genetically uniform clones for sustainable use and thereby saving the genetic diversity of this species in nature is important. In this regard, plant biotechnological applications such as micropropagation, synthetic seed production, and hairy root technology can play a significant role in a holistic conservation strategy. In addition to micropropagation, storage of these valuable genetic resources is equally important for germplasm preservation. However, more advanced research is warranted to determine the activities of bioactive compounds *in vitro* and *in vivo*, establish their underlying mechanisms of action and commence the process of clinical research.

## Introduction

One of the prerequisites for the success of primary health care is the availability and use of suitable drugs. Traditional medicine is still the most affordable and easily accessible source of treatment in the primary healthcare system. Medicinal plants have always been a potential source to cure different diseases, either in the form of traditional preparations or as pure active principles, and they are frequently the only source of medicine for the majority of people in the developing world.

**Figure 1:**
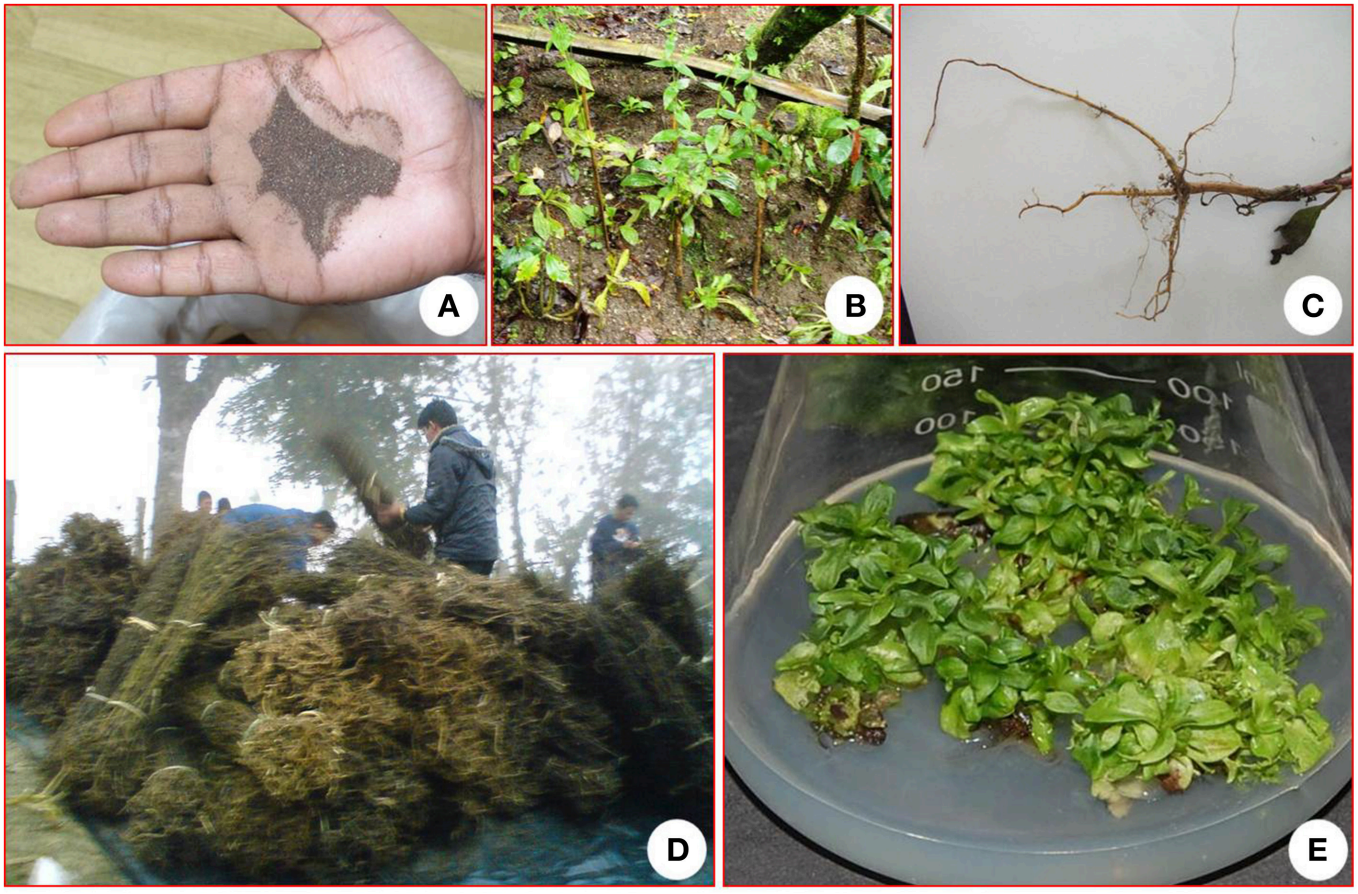
***Swertia chirayita.* (A)** Seeds, **(B)** Plant in nature, **(C)** Root of a mature plant, **(D)** Dry plant material, **(E)** High shoot multiplication in a plant tissue culture system.

**Figure 2:**
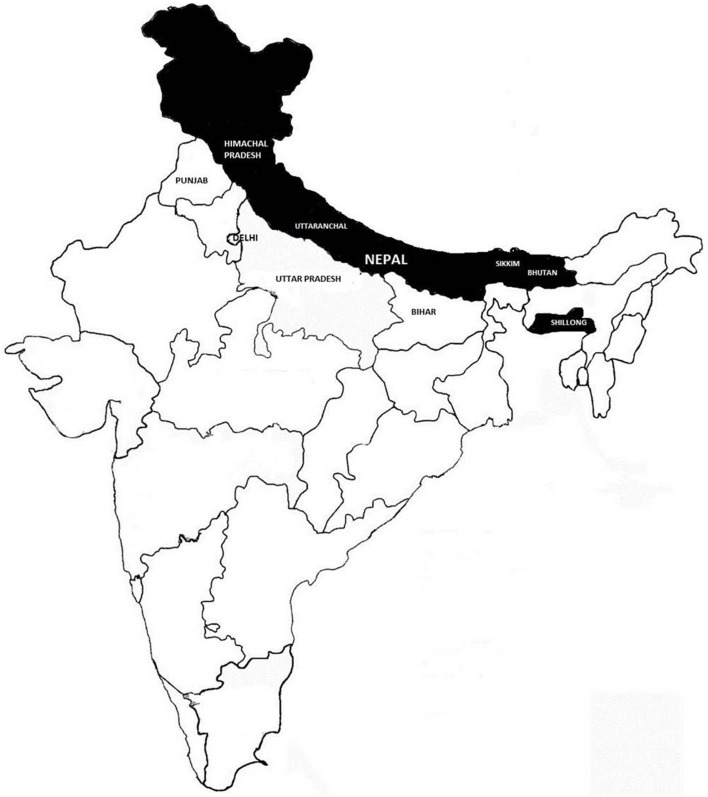
**Natural distribution of *Swertia chirayita.*** The shaded area represents the natural habitat of *Swertia chirayita* in the Himalayan Region.

*Swertia*, a genus in the family Gentianaceae include a large group of annual and perennial herbs, representing approximately 135 species. *Swertia* species are common ingredients in a number of herbal remedies. In India, 40 species of *Swertia* are recorded (Clarke, 1885; Kirtikar and Basu, 1984), of which, *Swertia chirayita* is considered the most important for its medicinal properties. *S. chirayita* was first described by Roxburgh under the name of *Gentiana chyrayta* in 1814 (Scartezzini and Speroni, 2000). *S. chirayita*, common name: “Chiretta” (Figure 1) is a critically endangered medicinal herb that grows at high altitudes in the sub-temperate regions of the Himalayas between 1200 and 2100 m altitudes from Kashmir to Bhutan (Bentley and Trimen, 1880; Clarke, 1885) on the slopes of moist shady places (Gaur, 1999; Figure 2). Its widespread uses in traditional medicine have resulted in over-exploitation from the natural habitat and it is now on the verge of extinction in the wild. *S. chirayita* is also known by an array of names such as Anaryatikta, Bhunimba, Chiratitka, Kairata in Sanskrit, Qasabuzzarirah in Arab and Farsi, Chiaravata in Urdu, Sekhagi in Burma, and Chirrato or Chiraita in Nepal (Joshi and Dhawan, 2005). Some authors have described *S. chirayita* as an annual (Anon, 1982; Kirtikar and Basu, 1984) and others as a biennial or pluri-annual (Edwards, 1993). This ethnomedicinal herb is known mostly for its bitter taste caused by the presence of different chemical constituents such as amarogentin (most bitter compound isolated till date), swerchirin, swertiamarin, and other bioactive compounds that are directly associated with human health welfare (Joshi and Dhawan, 2005). Due to its excessive over-exploitation from the natural habitat, narrow geographic occurrence (Bhat et al., 2013) and unresolved inherent problems of seed viability and seed germination (Badola and Pal, 2002; Joshi and Dhawan, 2005), alternative approaches for propagation and conservation are urgently required to avoid the possible extinction of this important species. Consequently, *S. chirayita* has been receiving increasing attention from a wide range of researchers as evident from the number of publications appearing in the literature (Chen et al., 2011; Nagalekshmi et al., 2011; Ghosh et al., 2012; Kumar and Chandra, 2013, 2014, 2015; Fan et al., 2014; Kumar et al., 2014; Sharma et al., 2014, 2015; Padhan et al., 2015; Zhou et al., 2015). However, a comprehensive review detailing the documented ethnomedicinal uses, pharmacological properties and safety evaluation carried out on *S. chirayita* and identifying the existing knowledge gap is lacking. In this review, we document the medicinal uses and phytochemical properties of *S. chirayita*. Future prospects including the potential conservation approaches to ensure a continuous supply for both local and international expanding markets and safety evaluation on uses of the species for medicinal purposes are highlighted.

### Botanical description

*S. chirayita* is an annual/biennial herb 0.6–1.5 m tall. It has an erect, around 2–3 ft long stem, the middle portion is cylindrical, while the upper is quadrangular, with a prominent decurrent line at each angle. Its stem is orange brown or purplish in color with large continuous yellowish pith (Bentley and Trimen, 1880; Joshi and Dhawan, 2005). Leaves are lanceolate, in opposite pairs, no stalks, acuminate, cordate at the base, sessile, five to seven nerved and 4 cm long (Scartezzini and Speroni, 2000). The root is simple, yellowish, somewhat oblique, or geniculate, tapering and short, almost 7–8 cm long and usually half an inch thick (Bentley and Trimen, 1880; Scartezzini and Speroni, 2000). Flowers are small, numerous, tetramerous, large leafy panicles, green-yellow, and tinged with purple and green or white hairs (Scartezzini and Speroni, 2000; Joshi and Dhawan, 2005). The calyx is gamophyllous with four lobes, corolla-lobes four twisted and superimposed, united at the base where they have pairs of nectaries on each lobe covered with long hairs. Stamens 4, opposite the corolla lobe, at the base of the corolla. Ovary unilocular with ovules laminal placentation parietale; two stigmas. Capsules are egg-shaped, 2-valved with a transparent yellowish pericarp. Seeds are numerous, very small and dark brownish in color (Chandra et al., 2012). Multi-colored corolla and the presence of nectaries support cross-pollination in *S. chirayita*.

## Medicinal uses

*S. chirayita* a traditional Ayurvedic herb is used by different indigenous population groups in multiple ways for several medicinal purposes (Table 1). The whole plant is widely used by local people for the treatment of hepatitis, inflammation, and digestive diseases (Bhatt et al., 2006). The wide range of medicinal uses include the treatment of chronic fever, malaria, anemia, bronchial asthma, hepatotoxic disorders, liver disorders, hepatitis, gastritis, constipation, dyspepsia, skin diseases, worms, epilepsy, ulcers, scanty urine, hypertension, melancholia, and certain types of mental disorders, secretion of bile, blood purification, and diabetes (Karan et al., 1999; Banerjee et al., 2000; Rai et al., 2000; Saha et al., 2004; Chen et al., 2011). Recently, *S. chirayita* extracts showed anti-hepatitis B virus (anti-HBV) activities (Zhou et al., 2015). Traditionally, decoctions of this species are used for anthelmintic, hepatoprotective, hypoglycemic, antimalarial, antifungal, antibacterial, cardiostimulant, antifatigue, anti-inflammatory, antiaging, antidiarrheal, as protectant of the heart and also help in lowering blood pressure and blood sugar (Schimmer and Mauthner, 1996). Herbal formulations such as Ayush-64, Diabecon, Mensturyl syrup, and Melicon V ointment (Edwin and Chungath, 1988; Mitra et al., 1996) contain *S. chirayita* extract in different concentrations for its antipyretic, hypoglycaemic, antifungal, and antibacterial properties. Furthermore, the curative value of this herb has also been recorded in ancient Ayurveda medicine systems and other conventional medical systems.

**Table 1:** **Ethnobotanical uses of *Swertia chirayita* in traditional medicine**.

**Plant part used**	**Traditional uses**	**References**
Whole plant	Used in several traditional and indigenous systems of medicines, such as Ayurveda, Unani, and Siddha	Mukherji, 1953; Kirtikar and Basu, 1984; Joshi and Dhawan, 2005;
Whole plant	Used in British and American pharmacopeias as tinctures and infusions	Joshi and Dhawan, 2005
Root	Serves as a drug and an effective tonic for general weakness, fever, cough, joint pain, asthma, and the common cold	Kirtikar and Basu, 1984; Joshi and Dhawan, 2005
Whole plant	For headaches and blood pressure, the leaves and chopped stems are soaked overnight in water. A paste is prepared and filtered with 1 glass of water. The preparation is consumed once a day for 2–3 days	de Rus Jacquet et al., 2014; Malla et al., 2015
Whole plant	For Tremor fever, whole *S. chirayita* plants are cut into small pieces and boiled in 1/2 L of water until the volume is reduced to less than half glass. The filtered water is stored in a glass bottle and half spoon is given to children once a day for 2 days. For adult, the posology is 1 spoon once in a day for 2 days and varies to three times a day until cured	de Rus Jacquet et al., 2014
Whole plant	Boiled in water and one cup of decoction is taken orally to cure malaria	Shah et al., 2014
Whole plant	Paste of the plant is applied to treat skin diseases such as eczema and pimples	Joshi and Dhawan, 2005; Malla et al., 2015
Whole plant	Liver disorders; stomach disorders like dyspepsia and diarrhea, intestinal worms	Mukherji, 1953; Joshi and Dhawan, 2005
Whole plant	Hiccups and vomiting, ulcers, gastrointestinal infections, and kidney diseases	Kirtikar and Basu, 1984
Whole plant	Used in combination with other drugs in cases of scorpion bite	Nandkarni, 1976
Whole plant	Used in excessive vaginal discharge	Jadhav and Bhutani, 2005

The widespread uses of *S. chirayita* in traditional drugs have resulted in considerable chemical analysis of the plant, and active principles which attribute the plant its medicinal properties. *S. chirayita* is also used in British and American pharmacopeias as tinctures and infusions (Joshi and Dhawan, 2005). The whole plant is used in traditional remedies but the root is mentioned to be the most bioactive part (Kirtikar and Basu, 1984).

## Pharmacological activity

The varied ethnobotanical uses of *S. chirayita* have led to the initiation of various pharmacological investigations. Previous research demonstrates that the *S. chirayita* extracts exhibit a wide range of biological activities, such as antibacterial, antifungal, antiviral, anticancer, anti-inflammatory, and others like antidiabetic and antioxidant activities (Verma et al., 2008; Alam et al., 2009; Arya et al., 2011; Chen et al., 2011; Laxmi et al., 2011). Concurrently, a diverse range of *in vitro* and *in vivo* test systems has been used to evaluate the pharmacological properties of *S. chirayita*. Evidence-based laboratory investigations indicate that aqueous, alcoholic and methanolic extracts of *S. chirayita* possess a number of promising pharmacological properties. The whole plant of *S. chirayita* have been reported to be used for the treatment of antibacterial and antifungal activity (Alam et al., 2009; Laxmi et al., 2011; Rehman et al., 2011). Anti-hepatitis B virus activity of *S. chirayita* extracts was also studied on HepG 2.2.15 cells line (Zhou et al., 2015). The whole plant of *S. chirayita* has been reported for the anti-inflammatory and hypoglycemic activity (Banerjee et al., 2000; Kar et al., 2003; Alam et al., 2011; Das et al., 2012; Verma et al., 2013). Chen et al. (2011) investigated the 70% ethanolic extract of *S. chirayita* for antioxidant activities by using antioxidant tests including reducing power and beta-carotene assay. The results showed that 70% ethanolic extracts exhibited high DPPH scavenging activity (IC_50_ = 267.80 μg/mL). Table 2 presents a summary focusing on the pharmacological evaluations using *in vitro* and *in vivo* systems whereas Table 3 provides antioxidant potential of *S. chirayita*.

**Table 2:** **Evaluation of the biological activities of *Swertia chirayita***.

**Bioactivity evaluated**	**Plant part(s) tested**	**Test system**	**^a^Extracting solvent**	**Test Organism/Models**	**Control**	**Toxicity test**	**References**
Antibacterial	Whole plant	*In vitro*	EtOH	*Escherichia coli* ATCC 26922	Ciprofloxacin	None	Rehman et al., 2011
				*Klebsiella pneumonia* ATCC 15380			
				*Pseudomonas aeruginosa* ATCC 25619			
				*Proteus vulgaris* ATCC 6380			
Antibacterial	Stem	*In vitro*	MeOH	*Bacillus subtilis* ATCC 6633	Ceftriaxone, Ceftriaxone sodium, Cefuroxine, Ciprofloxacin, Gentamycine, Levofloxacin, Metronidazole, Tranexamicacid	None	Khalid et al., 2011
				*Enterococcus faecalis* (ATCC 14506)			
				*Staphylococcus aureus* (ATCC 6538)			
				*Pseudomonas aeruginosa* (ATCC 27853)			
				*Salmonella typhi* (ATCC 14028)			
Antibacterial	Whole plant	*In vitro*	MeOH	*Bacillus subtilis* MTCC 736	Gentamycin	None	Laxmi et al., 2011
				*Bacillus polymyxa*			
				*Staphylococcus aureus* MTCC 3160			
				*Escherichia coli* MTCC 723			
				*Salmonella typhi* MTCC 3216			
				*Vibria cholera* MTCC 3906			
				*Streptococcus pyogenes* MTCC 1927			
				*Proteus mirabilis* MTCC 1429			
				*Providentia alkalifaciens*			
				*Pseudomonas aeruginosa* MTCC 7837			
Antibacterial	Whole plant	*In vitro*	DCM; EtOH	*Staphylococcus aureus*	Kanamycin 30 μg/disc	None	Alam et al., 2009
Antibacterial	Stem	*In vitro*	EtOH	*Staphylococcus aureus*	Chloramphenicol 30 μg/disc	Brine shrimp assay–positive	Sultana et al., 2007
				*Bacillus subtilis*			
				*Salmonella typhi*			
				*Shigella flexeneriae*			
				*Sarcina lutea*			
				*Bacillus megaterium*			
Antifungal	Whole plant	*In vitro*	MeOH	*Aspegillus niger* MTCC 1881	Amphotericin	None	Laxmi et al., 2011
				*Aspergillus flavus* MTCC 1883			
				*Cladosporium oxysporum* MTCC 1777			
Antileishmanial	Aerial part	*In vitro*	95% EtOH	*Leishmania donovani UR6*	–	None	Ray et al., 1996
Antileishmanial	Whole plant	*In vitro*	MeOH	*Leishmania donovani AG83*	–	Cytotoxicity test-negative	Medda et al., 1999
Antihelmintic	Whole plant	*In vitro*	Water; MeOH	*Haemonchus contortus*	Levamisole 0.55 mg/ml	None	Iqbal et al., 2006
Antimalarial	Leaves/Stem	*In vitro*	MeOH; PE; Water; EtOH	*Plasmodium falciparum* FCK 2	Parasitized red blood cells and 10 μCi of [^35^S]-methionine	None	Bhat and Surolia, 2001
Egg hatchability and larvicidal	Whole plant	*In vitro*	HEX; EA; MeOH	*Aedes aegypti*	Tween-80	None	Balaraju et al., 2009b
				*Culex quinquefasciatus*			
Anti-hepatitis B virus	Whole plant	*In vitro*	50% EtOH	HepG 2.2.15 cells line	Tenofovir	None	Zhou et al., 2015
Antiinflammatory	Aerial parts	*In vivo*	Petroleum	N/A	Mice treated with vehicle or Diclofenac (10 mg/kg)	None	Banerjee et al., 2000
Antiinflammatory	Root	*In vivo*	95% EtOH	N/A	Diclofenac (25 mg/kg)	None	Das et al., 2012
Hypoglycemic	Whole plant	*In vivo*	95% EtOH	N/A	Mice treated with vehicle	None	Kar et al., 2003
Hypoglycemic	Leaves	*In vivo*	EtOH	N/A	Glibenclamide (5 mg/kg)	None	Alam et al., 2011
Hypoglycemic	Whole plant	*In vivo*	EA; EtOH	N/A	Glibenclamide (5 mg/kg)	Cytotoxicity test-negative	Verma et al., 2013
Antidiabetic	Whole plant	*In vitro*	95% EtOH; HEX	STZ-NAD(streptozotocin- nicotinamide) induced diabetic albino mice	Metformin (100 μg/kg)	None	Grover et al., 2002
Antidiabetic	Whole plant	*In vitro*	EtOH; HEX; Chloroform	STZ-NAD(streptozotocin- nicotinamide) induced diabetic albino mice	Metformin (100 μg/kg)	None	Arya et al., 2011
Antipyretic	Root	*In vitro*	Water	Brewer's yeast induced pyrexia Typhoid-Paratyphoid A, B vaccine induced Hyperexia	Paracetamol (150 mg kg^−1^)	None	Bhargava et al., 2009
Anticarcinogenic	Whole plant	*In vivo*	HEX	N/A	9,10-dimethyl benz(a)anthracene (DMBA)	None	Saha et al., 2004
Analgesic	Leaves/Stem	*In vivo*	EtOH	N/A	Diclofenac sodium (25 mg/kg)	None	Alam et al., 2010
Analgesic	Root	*In vivo*	EtOH	N/A	Aminopyrine (50 mg/kg)	None	Das et al., 2012
Hepatoprotective	Aerial parts	*In vivo*	70% EtOH	N/A	Paracetamol (150 mg/kg)	None	Nagalekshmi et al., 2011
CNS	Whole plant	*In vivo*	EtOH	N/A	Mice treated with vehicle	None	Bhattacharya et al., 1976
Antiviral	Leaves/Stem	*In vitro*	Water	Herpes simplex virus type-1	Acyclovir (1 mg/mL)	Cytotoxicity test-negative	Verma et al., 2008

a Extracting solvent: EtOH, ethanol; EA, ethyl acetate; HEX, hexane; MeOH, methanol; N/A, not applicable; PE, petroleum ether.

**Table 3:** **Antioxidant potential of different solvent extracts of *S. chirayita***.

**Plant part tested**	**^a^Extracting solvent**	**Test system**	**Control used and result**	**Toxicity test**	**References**
Whole plant	70% EtOH	*In vitro*	BHT and Vitamin C	None	Chen et al., 2011
			IC_50_ = 267.80 μg/mL (DPPH)		
			IC_50_ = 1.502 ± 0.200 μg/mL (β-carotene)		
			IC_50_ = 6.50 μg/mL (ABTS)		
Whole plant	70% EtOH	*In vivo*	NA	Cytotoxicity test-negative	Chen et al., 2011
Whole plant	MeOH	*In vitro*	BHT	None	Sharma et al., 2013b
			EC_50_ = 27.70 μg/ml (DPPH)		
Whole plant	MeOH	*In vitro*	BHA	None	Ahirwal et al., 2014
			IC_50_ = 222.74 μg/mL (DPPH)		
Whole plant	Water	*In vitro*	Gallic acid	None	Kumar et al., 2011
			EC_50_ = 315.83 μg/mL (DPPH)		
Leaves	Water	*In vitro*	BHA; BHT	None	Ghosh et al., 2012
			IC_50_ = 86 μg/mL (DPPH)		
			900 ± 11(4 min) and 2070 ± 110 (30 min) μM Fe (II)/g sample DW (FRAP)		
Whole plant	12% EtOH	*In vitro*	Ascorbic acid	None	Phoboo et al., 2013
			IC_50_ = 156.62 μg/mL (DPPH)		
Whole plant		*In vitro*	Gallic acid	None	Kshirsagar et al., 2015
	MeOH		IC_50_ = 551.26 μg/mL (DPPH)		
	EtOH		IC_50_ = 557.61 μg/mL (DPPH)		
	ACE		IC_50_ = 551.96 μg/mL (DPPH)		
	Water		IC_50_ = 559.05 μg/mL (DPPH)		

ABTS, 2,2-azino-bis (3-ethylebenzthiazoline-6-sulphonicacid); BHA, Butylated hydroxy anisole; BHT, Butylated hydroxytoluene; DPPH, 2,2-Diphenyl-1-picrylhdrazyl; DW, Dry weight; FRAP, Ferric Reducing Antioxidant Power

a Extracting solvent: ACE, acetone; EtOH, ethanol; MeOH, methanol

## Phytochemistry

**Figure 3:**
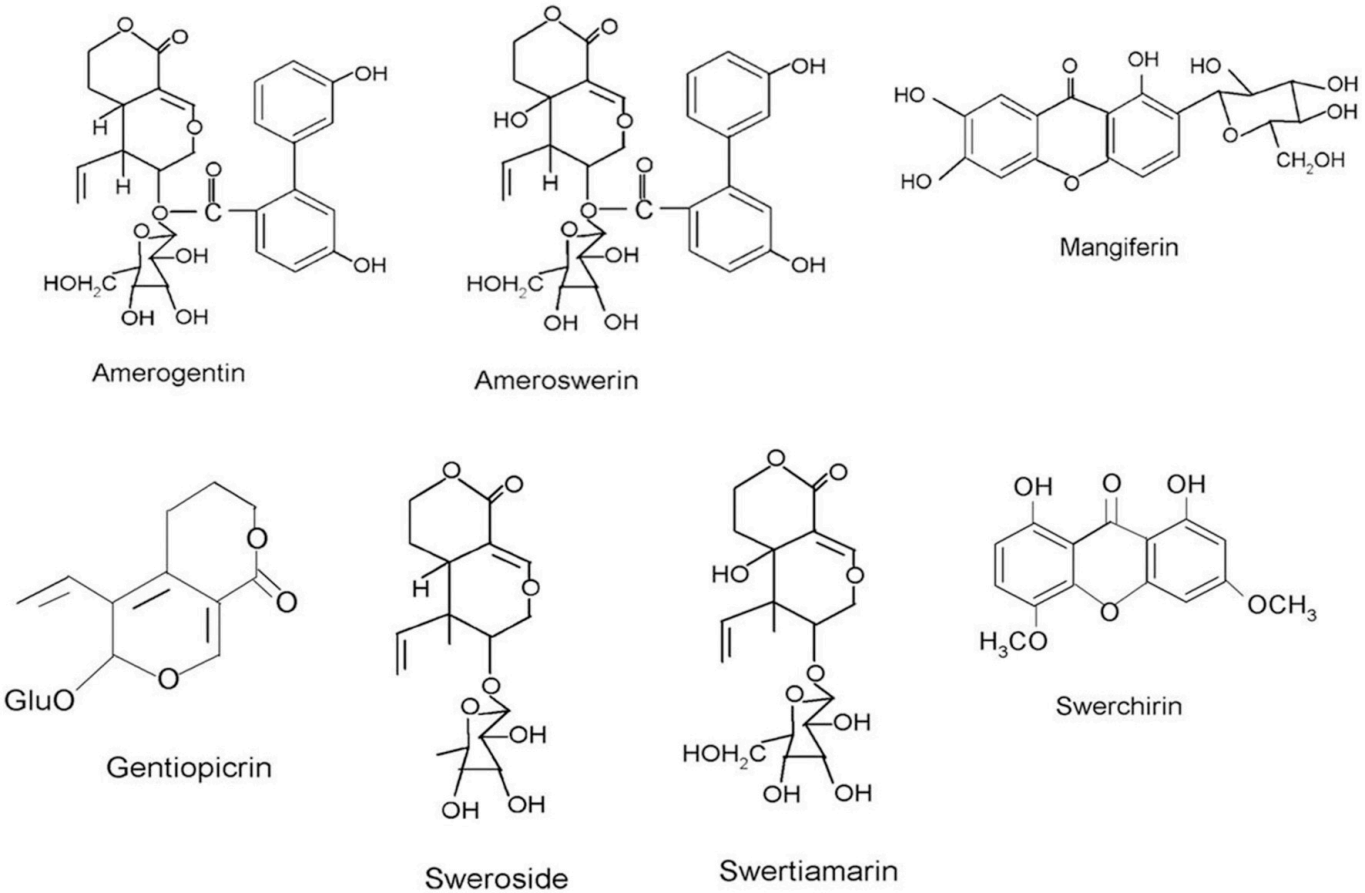
**Chemical structures of important phytoconstituents found in *Swertia chirayita***.

The widespread uses of *S. chirayita* as a traditional drug and its commercialization in modern medical systems have led to a rise in scientific exploration of its phytochemistry in order to identify the active phytochemicals. This has resulted in a considerable body of literature exploring the chemical constituents of this plant (Mandal and Chatterjee, 1987; Chakravarty et al., 1991, 1994; Mandal et al., 1992; Chatterjee and Pakrashi, 1995; Pant et al., 2000). The wide-range biological activities of *S. chirayita* are attributed to the presence of a diverse group of pharmacologically bioactive compounds belonging to different classes such as xanthones and their derivatives, lignans, alkaloids, flavonoids, terpenoids, iridoids, secoiridoids, and other compounds such as chiratin, ophelicacid, palmitic acid, oleic acid, and stearic acid (Pant et al., 2000; Patil et al., 2013). The first isolated dimeric xanthone was chiratanin present in different parts of *S. chirayita*. The pharmacological efficacy of *S. chirayita* has been partly attributed to the biological activity of major phytoconstituents including amarogentin, swertiamarin, mangiferin, swerchirin, sweroside, amaroswerin, and gentiopicrin (Figure 3). Amarogentin is reported to be anti-diabetic (Phoboo et al., 2013), anticancerous (Saha et al., 2006; Pal et al., 2012), and antileishmanial (Ray et al., 1996; Medda et al., 1999), whereas swertiamarin has been tested for its anti-hepatitis (Wang et al., 2001), anticancer (Kavimani and Manisenthlkumar, 2000), anti-arthritic activities (Saravanan et al., 2014). It has been shown to exhibit anti-diabetic (Vaidya et al., 2013) properties. Mangiferin is also reported to have anti-diabetic, antiatherosclerotic (Pardo-Andreu et al., 2008), anticancer, anti-HIV (Guha et al., 1996), antiparkinson (Kavitha et al., 2013), and chemopreventive (Yoshimi et al., 2001) activities. Swerchirin is known to be antimalarial, hypoglycemic (Bajpai et al., 1991; Saxena et al., 1996), hepatoprotective, pro-heamatopoietic (Ya et al., 1999), with blood glucose lowering activity (Sekar et al., 1987; Saxena et al., 1991) and weak chemo preventive pharmacological effects (Hirakawa et al., 2005). Swerchirin at different concentrations (1, 10, and 100 μM) significantly enhanced glucose stimulated insulin release from isolated islets (Saxena et al., 1993). Sweroside is reported to be antibacterial (Siler et al., 2010), hepatoprotective (Liu et al., 1994; Luo et al., 2009), preventative in treatment for hyperpigmentation (Jeong et al., 2015), and is also suggested as a promising osteoporosis therapeutic natural product (Sun et al., 2013). Amaroswerin is known for its gastroprotective effects of the bitter principles (Niiho et al., 2006). Table 4 provides a summary focusing on the biological activity of the phytochemicals present in *S. chirayita*.

**Table 4:** **Important bioactive compounds isolated from *Swertia chirayita***.

**Phytochemical**	**Biological activity**	**References**
Amarogentin	Antileishmanial	Ray et al., 1996; Medda et al., 1999
	Topoisomerase inhibitor	Ray et al., 1996
	Anticancer	Saha et al., 2006; Pal et al., 2012
	Anti-diabetic	Phoboo et al., 2013
	Gastroprotective	Niiho et al., 2006
Swertiamarin	CNS depressant	Bhattacharya et al., 1976
	Anticholinergic	Suparna et al., 1998
	Anticancer	Kavimani and Manisenthlkumar, 2000
	Anti-hepatitis	Wang et al., 2001
	Antibacterial	Kumarasamy et al., 2003
	Cardio-protective, anti-atherosclerotic	Vaidya et al., 2009
	anti-diabetic	Vaidya et al., 2013
	Anti-arthritic	Saravanan et al., 2014
Mangiferin	Antiviral	Zheng and Lu, 1990
	Immunomodulatory, antitumor, anti-HIV	Guha et al., 1996
	Antioxidant	Sanchez et al., 2000
	Chemopreventive	Yoshimi et al., 2001
	Antiinflammatory	Kumar et al., 2003
	Hypoglycemic	Muruganandan et al., 2005
	Anti-diabetic, Antiatherosclerotic	Pardo-Andreu et al., 2008
	Antiparkinson	Kavitha et al., 2013
Swerchirin	Hypoglycemic	Bajpai et al., 1991; Saxena et al., 1996
	Hepatoprotective, pro-heamatopoietic	Ya et al., 1999
	Blood glucose lowering activity	Sekar et al., 1987; Saxena et al., 1993
	Chemopreventive	Hirakawa et al., 2005
Sweroside	Antibacterial	Siler et al., 2010
	Hepatoprotective	Liu et al., 1994; Luo et al., 2009
	Hyperpigmentation	Jeong et al., 2015
	Osteoporosis	Sun et al., 2013
Amaroswerin	Gastroprotective	Niiho et al., 2006
Gentianine	Antipsychotic	Bhattacharya et al., 1974
	Antimalarial	Natarajan et al., 1974
Oleanolic acid	Antimicrobial	Jesus et al., 2015
	Antitumor	Soica et al., 2014
	Antiinflamatory, antioxidant	Liu, 1995
Ursolic acid	Antimicrobial	Jesus et al., 2015
	Antitumor	Bonaccorsi et al., 2008; Soica et al., 2014
Swertanone	Antiinflammatory	Kumar et al., 2003; Tabassum et al., 2012
Syringaresinol	Hepatoprotective	Chakravarty et al., 1994
Bellidifolin	Hypoglycemic	Basnet et al., 1995
Isobellidifolin	Hypoglycemic	Basnet et al., 1995
1-Hydroxy-3,5,8-trimethoxyxanthone	Antimalarial	Mandal and Chatterjee, 1994
1-Hydroxy-3,7,8-trimethoxyxanthone	Spasmogenic agent	Ateufack et al., 2007
	Antiulcerogenic	Ateufack et al., 2014
1,5,8-trihydroxy-3-methoxyxanthone	Blood sugar lowering	Ghosal et al., 1973
β-Amyrin	Anti-inflammatory	Holanda et al., 2008
	Antimicrobial, antifungal	Vázquez et al., 2012
Chiratol	Anti-inflammatory	Banerjee et al., 2000

## Safety evaluation

Concerns regarding safety of conventional drugs are vital issues of pharmaceutical industries. Studies have indicated that some commonly used medicinal plants may be mutagenic or cytotoxic especially over a long period of use (Verschaeve and Van Staden, 2008). There is increasing evidence on the toxicity of crude extracts and isolated compounds from different plant species (Koorbanally et al., 2006). However, despite its long history of use in traditional medicine, there is still a lack of scientific information concerning the safety evaluation of *S. chirayita*. It can be traced through the medicinal history as a nontoxic and safe ethnomedicinal herb and has been mentioned in medical papyri to expel fever, relieve headache, inflammation, and to stimulate the central nervous system. *S. chirayita* extracts, did not cause obvious toxic effects in mice as there were no significant differences in body weight and body temperature between the treated and control groups (Alam et al., 2011; Das et al., 2012). A clinical study by Medda et al. (1999) concluded that *S. chirayita* revealed no evidence of toxicity in both liposomal and niosomal forms. Furthermore, stringent efforts are required to further delineate the well-documented toxicological properties involving toxicity and mutagenic tests to evaluate the safety of this plant. Nevertheless, rigorous clinical studies involving different mechanisms are still needed to confirm the safety of *S. chirayita* in traditional medicine so that it can be used safely and effectively. Despite the fact that the benefits of medicinal plants is globally acknowledged, the need for better insight on the safety evaluation remains essential, so as to differentiate between toxic effects and pharmacological importance of plant extracts (Aremu and Van Staden, 2013).

## Swertia chirayita conservation

Destruction of plant resources is a normal occurrence. The current speed of extinction through human interferences is estimated to be approximately 100–1000 times faster than the natural speed of extinction (Chapin et al., 2000). Due to developmental activities in the Himalayan region, wild populations of many medicinal plants, including *S. chirayita* are reduced to the verge of extinction. *S. chirayita* is traded and used mostly as a traditional drug. Due to its multiple uses the demand is on the rise by both national and international trading leading to increasing over harvesting of wild populations. This has resulted in drastic reductions of its populations. Lack of comprehensive data on annual harvested and traded plants of *S. chirayita* is also a major concern. According to the International Union of Conservation of Nature (IUCN) criteria, *S. chirayita* conservation status has been categorized as “critically endangered” (Joshi and Dhawan, 2005). *S. chirayita* is among the 32 most highly prioritized medicinal plants of India as identified by The National Medicinal Plant Board, Government of India (http://www.nmpb.nic.in).

The implication of losing this plant species due to extinction lies not only in the loss of genes useful for plant development or in the biosynthesis of new compounds but also the loss of potentially novel compounds of pharmaceutical or nutraceutical benefit. In order to meet the escalating demand in national and international trade markets of raw plants, cultivation must be escalated. There are limitations in the use of seed propagation, due to low viability, and low germination percentages (Badola and Pal, 2002; Chandra et al., 2012). Biotechnology offers new means of improving biodiversity and biotechnological approaches such as micropropagation techniques (Figure 1E) has received more attention and may play a vital role in the establishment of genetically uniform plants for the *Swertia* industry. It is believed that the development of efficient micropropagation protocols, can guarantee an adequate supply of *S. chirayita* plants (devoid of environmental-imposed constraints) with subsequent reduction in uncontrolled harvesting pressure on wild populations. Several studies reported on micropropagation, somatic embryogenesis and acclimatization procedures with the capacity to produce many uniform *S. chirayita* clones throughout the year (Kumar and Chandra, 2013, 2014; Kumar et al., 2014). As shown in Table 5, micropropagation protocols have successfully been established for *S. chirayita* using different explants.

**Table 5:** **Micropropagation data for *Swertia chirayita***.

**Tissue culture Study**	**Explant type**	**Optimum concentrations**	**Major observations**	**References**
Regeneration	Seeds	3.0 μM BA	Adventitious shoot regeneration from root explants	Wawrosch et al., 1999
Micropropagation	*In vivo* axillary bud/shoot apices	0.5 mg/l BA + 1.0 mg/l GA_3_	Methods and compositions for rapid *in vitro* propagation	Ahuja et al., 2003
Axillary multiplication	Seedling-derived nodal explants	4.0 μM BA + 1.5 μM 2iP	Improved shoot proliferation	Joshi and Dhawan, 2007
Regeneration	*In vivo* stem with node	0.44 μM BA + 4.65 μM KN	Improved regeneration from the nodal explants	Chaudhuri et al., 2007
Direct shoot multiplication	*In vitro* leaves	2.22 μM BA + 11.6 μM KN + 0.5 μM NAA	Improved protocol for propagation	Chaudhuri et al., 2008
Regeneration	Seeds	2.22 μM BA + 2.22 μM KN + 0.54 μM NAA	Regeneration from immature seed culture	Chaudhuri et al., 2009
Direct shoot regeneration	*In vivo* leaves	13.32 μM BA + 0.54 μM NAA	*In vitro* shoot regeneration	Wang et al., 2009
Micropropagation	*In vitro* shoot tip	1.0 mg/l BA + 0.1 mg/l KN	Improved shoot proliferation	Balaraju et al., 2009a
*In vitro* regeneration	Node	2 mg/l BA	Rapid *in vitro* propagation system	Koul et al., 2009
Shoot Organogenesis	*In vitro* root	4.44 μM BA + 1.07 μM NAA	Improved protocol for plant regeneration	Pant et al., 2010
Somatic embryogenesis	*In vivo* leaves	1.0 mg/l 2,4-D and 0.5 mg/l 2,4-D + 0.5 mg/l BA	Rapid system for micropropagation	Balaraju et al., 2011
Callus culture	*In vitro* root	13.32 μM BA + 0.90 μM 2,4-D	Plant regeneration via indirect organogenesis	Pant et al., 2012
Efficient Regeneration	*In vivo* shoot tip	0.5 mg/l BA + 1.0 mg/l GA_3_	An efficient shoot proliferation	Kumar and Chandra, 2013
*In vitro* flower production	Axillary bud	1.0 mg/l BA + 70 mg/l Adenine sulfate	*In vitro* flowering and effective protocol for regeneration	Sharma et al., 2014
Somatic embryogenesis	*In vivo* leaves	0.5 mg/l 2,4-D + 0.5 mg/l KN	An efficient protocol for plant regeneration through somatic embryogenesis	Kumar and Chandra, 2014
Direct and Indirect regeneration	*In vivo* leaves	1.0 mg/l BA + 100 mg/l Adenine sulfate + 0.1 mg/l IAA	An efficient protocol of plant regeneration through direct and indirect organogenesis	Kumar et al., 2014

2,4-D, 2,4-Dicholorophenoxyacetic acid; BA, 6-benzyl-adenine; GA_3_, Gibberellic acid; IAA, Indole-3-acetic acid; KN, Kinetin; NAA, Naphthalene Acetic Acid.

Synthetic seed technology is also an applied application of modern plant biotechnology which offers tremendous potential for easy handling, micropropagation and plant germplasm conservation through cryopreservation (Ara et al., 2000; Sharma et al., 2013a; Perveen and Anis, 2014; Gantait et al., 2015). Successful implementation of synthetic seed technology for mass propagation and short-term storage of genetically uniform clones require manipulation of *in vitro* tissue culture systems that are able to transform into complete plantlets (Ara et al., 2000). Recently, Kumar et al. (2014) reported on synthetic seed production and plant regeneration of *S. chirayita* from somatic embryos. However, further studies are required to improve technology so that it can be used on a commercial scale.

Many plant secondary metabolites accumulate in roots (Flores et al., 1999) but harvesting of these organs is destructive. Therefore, in the recent past *Agrobacterium rhizogenes* induced hairy root technology has received attention and engaged a new platform of applied research in generating pharmaceutical lead compounds. The large scaling-up of hairy root cultures is of importance for biotechnological applications (Guillon et al., 2006). Attempts have been made to standardize *A. rhizogenes* transformed root cultures for production of active secondary metabolites under *in vitro* conditions of *S. chirayita* (Keil et al., 2000). For commercialization of *S. chirayita* adventitious roots and to elucidate the feasibility for commercial application, hairy root technology is required along with various factors affecting the production of root biomass and bioactive compounds. Overall, micropropagation which is conducted under a controlled environment will help to prevent the current plant biodiversity conservation problems arising from over harvesting practices of wild populations and can profoundly improve the quality of bioactive secondary metabolites of this age old medicinal plant *S. chirayita*.

## Conclusions and future perspectives

*S. chirayita* offers many promising prospects for both traditional and modern medicine. *S. chirayita* is apparently a potential herbal therapy for many ailments. This review summarized the existing ethnobotanical uses, phytochemistry, pharmacological activities, safety evaluation, and conservation status on *S. chirayita*.

So far no serious side effects or toxicity of *S. chirayita* have been reported, but further toxicological studies are still needed to confirm the safety of *S. chirayita* in humans. Efforts are required for further studies, especially evaluating its biological activities *in vivo* and toxicological and mutagenic properties in order to better validate the safety of these different plant-derives compounds. In all probability there is a need for clinical trials to establish the efficacy of using *S. chirayita* in medicine. Due to its multiple uses the demand in both national and international markets is constantly on the rise. Overexploitation combined with habitat destruction has resulted in the drastic reduction of its population. For the successful commercialization of this critically endangered medicinal plant any proposed research must be viewed in a wider context that includes conservation practices and sustainable supply of raw plants. This will require innovative tools, which utilize biotechnological interventions, including micropropagation, cryopreservation, and bioreactors for the conservation, as well as for raising commercial production. In synthetic seed technology more detailed research is required mainly for improvement in germination frequency of synthetic seeds and subsequent plantlet growth in soil so that it can be used on a commercial scale. Additionally, in the near future, hairy root technology can be used as a model system and will also provide plant biotechnologists with powerful tools to improve the valuable phytochemicals of *S. chirayita*. Although efficient micropropagation protocols have been established, further studies focusing on seed biology and ways of improving bioactive secondary metabolites in cultivated *S. chirayita* would be beneficial for their commercialization. Quality control protocols to prevent misidentification and possible adulteration of *S. chirayita* are also needed. In summary, *S. chirayita* have been studied extensively in terms of taxonomy, ethnobotany, phytochemistry, biological activities, and conservation. However, new findings may increase the present therapeutic importance of *S. chirayita* and promote their future use in modern medicine, while novel biotechnological approaches are required for further conservation.

## Author contributions

VK conducted the research and wrote the paper. JVS supervised the work and proof read the paper.

### Conflict of interest statement

The authors declare that the research was conducted in the absence of any commercial or financial relationships that could be construed as a potential conflict of interest.

## Abbreviations

ABTS: 2,2-azino-bis (3-ethylebenzothiazoline-6-sulphonic acid)
ACE: Acetone
BA: 6-benzyl-adenine
BHA: Butylated hydroxy anisole
BHT: Butylated hydroxytoluene
2, 4-D: 2,4-Dicholorophenoxyacetic acid
DPPH: 2,2-diphenyl-1-picrylhydrazyl
DW: Dry weight
EtOH: Ethanol
EA: Ethyl acetate
FRAP: Ferric Reducing Antioxidant Power
GA_3_: Gibberellic acid
HEX: Hexane
IAA: Indole-3-acetic acid
KN: Kinetin
MeOH: Methanol
NAA: Naphthalene Acetic Acid
PE: Petroleum ether.
